# Extraction of *Rhododendron arboreum* Smith flowers from the forest for the livelihood and rural income in Garhwal Himalaya, India

**DOI:** 10.1038/s41598-021-00257-y

**Published:** 2021-10-21

**Authors:** D. S. Chauhan, Pankaj Lal, A. K. Shrama

**Affiliations:** 1 Department of Forestry and NR, HNB Garhwal University, Srinagar Garhwal, 246174 India; 2 NWFP Division, Forest Research Institute, Dehradun, India

**Keywords:** Ecology, Plant sciences

## Abstract

*Rhododendron arboreum* locally known as ‘Burans', that bears magnificent flowers is one of the valuable non timber forest produces (NTFPs) in Garhwal Himalaya. These flowers are good source of income for local populace and help them to their subsistence up to some extent. *R. arboreum* flower can help local population to improve their livelihoods if potential harvesting is carried out sustainably. An attempt has been made to estimate the flower yield, examine extraction techniques, marketing trends and various uses of flowers. Stratified random sampling method was carried out in eight sites varying in altitudes and geographic locations. Flower yield kg/ha for each site was calculated as standard process. Questionnaire based survey was carried out in selected villages for flower extraction and marketing trends. Projections of potential (probable/-could generate) income were made and cost–benefit analysis was also estimated. Tree density of *R. arboreum* ranked first and *Q. leucotrichophora* had second rank while 16–25 cm cbh class tree density for *R. arboreum* was found highest across the sites. Flower yield was significantly (p < 0.001) higher at Khirsu site with 26–35 and 46–55 cm cbh class. There was positively significant correlation (n = 446, p < 0.001, r = 0.53) between flower yield and actual cbh. Flower yield has a direct relation with size of tree whereas yield has been less impacted by the sites. Average yield of flowers across the sites was about 25.3 ton/ha. On average 30% households are engaged in the extraction and trade activities with the extraction rate of 25–350 kg/household/year. A net household income of Rs. 6000–37,000 (89–545 USD) per year was computed from *Rhododendron* flower extraction and marketing business. The total monetary benefit was significantly higher than the inputs for all value added items on a per day basis. *R. arboreum* plays important role in ecological and economic sustainability of poor rural people and unemployed youths in Himalayan region. This can reduce unemployment through development of small cottage industry and entrepreneurship at village level by making different food products i.e. juice, squash, sauce and pickle etc.

## Introduction

Non-timber forest produces (NTFPs) play vital role among the local people and provide a source of income and subsistence living^1,2^. In Western Himalaya, most of the NTFPs are integral part of day-to-day livelihood activities especially for rural population. The collection of NTFPs is a major economic activity and about 500 million people living in or near forests are depended upon them for meeting their livelihood needs^3^.

In Uttarakhand, NTFPs did not contribute more than 6% of the total income of any village^4^. It was noticed that people are quite cautious while speaking about the collection of NTFPs from the forests due to the fear of forest officials and hence earning from NTFPs is often under reported. There is a vibrant market for NTFPs in the region and most of the NTFPs are being traded through government agencies. Although these NTFPs are present throughout the state but are in abundance in areas above 2000 m altitude, or in villages with access to high altitude pastures. Roughly over 30% households depend on NTFPs for a substantive part of their earnings^4^.

*Rhododendron arboreum* locally called ‘Burans’ is small evergreen tree widely distributed between 1300 to 3500 m asl occurring from Western to Eastern Himalayan region and other neighboring countries^5^. In Western Himalaya it occurs mainly in association with *Quercus leucotrichophora* (Banj oak), *Pinus roxburghii* (Chir pine), *Myrica esculenta* and *Lyonia ovalifolia* in temperate forest and sub-alpine vegetation. The tree has high economic value as squash/juice and chutneys made out of its flowers are very popular throughout the state. The magnificent deep red or pale pink colour flowers bloom during January–March with sweet and sour taste which are used in the preparation of squash, jam, jelly and other local food products. Refreshing juice of *Rhododendron* flowers is a common and pleasant drink, serves as a refreshing cold drink during hot summer throughout the region. In addition to food products, Burans flower has various phytochemicals and therefore it is being utilized as home remedy for several diseases. Burans flower juice has antioxidant property and protect heart from oxidative stress and reduce risk of stroke and other heart disorders.

*Rhododendron arboreum* flower has emerged as one of the valuable NTFPs in the Garhwal Himalaya. Squash, juice, jam and jelly made from its flowers have a great potential for income generation. Present study was designed to quantify flower yield in potential resource areas to evaluate extraction/harvesting techniques and existing market trend as well as to estimate the cost–benefit analyses of various value added products.

## Materials and methods

### Study sites and sampling

**Figure 1:**
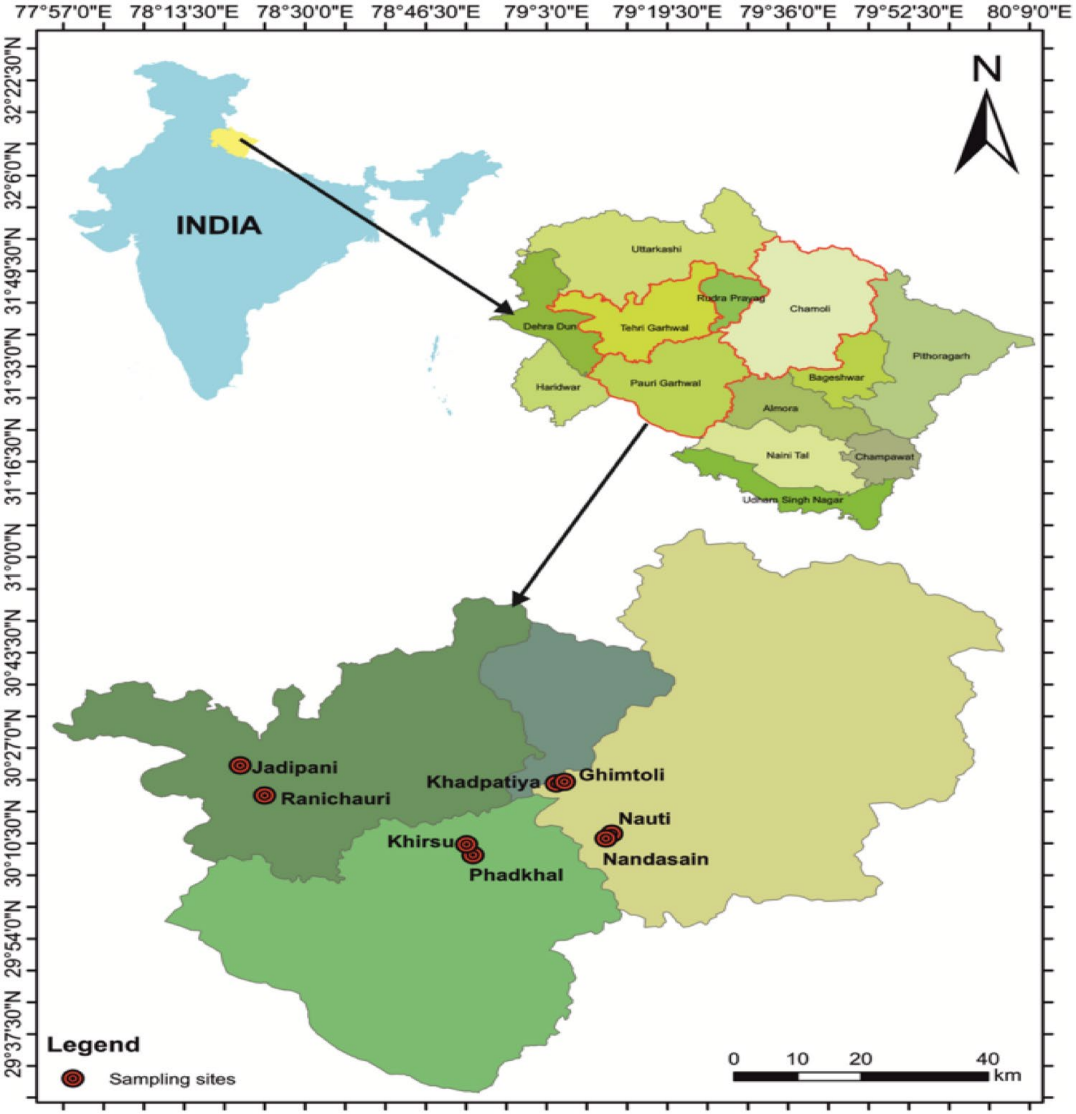
Locations of *Rhododendron arboreum* study sites in Garhwal region (ARC GIS software 10.5 version was used for map preparation. The map was created by Mr. Raman Patel, Research scholar, Dept. of Geology, HNB Garhwal University, Srinagar, Uttarakhand, India).

The study was conducted in Garhwal region (Western Himalaya) from 2016 to 2017 at eight *Rhododendron arboreum* rich areas in four hill districts (Chamoli, Tehri, Pauri and Rudraprayag). Voucher specimen of *Rhododendron arboreum* collected and have been deposited in the Herbarium, Botany department, HNB Garhwal University (specimen no. GUH 8510)^6^. Identification of *R. arboreum* has been done through A Field Guide book authored by Rai et al.^7^. Since it is a wild species and flowers have been collected for our research and field study under the permission from competent authority of State Forest Department, Govt. of Uttarakhand. According to IUCN’s Red List Categories and Criteria, globally *Rhododendron arboreum* comes under Least Concern (LC) category^8^. These sites are situated between 30°08′47″ to 30°24′06″ N latitude and 78°25′05″ to 79°12′39″ E longitude with altitudes from 1820 m asl in Nandasain and 2270 m asl in Jadipani (Table 1; Fig. 1). All sites were well stocked (mean stand density ≥ 500 tree/ha) with *Rhododendron arboreum* trees mixed with *Quercus leucotrichophora.* We referred these resource rich sites as *R. arboreum* habitats (Table 1). Stratified random sampling method (i.e. stand density and CBH class’s strata) were carried out these eight sites. Total sampled area 0.2 ha in each site; two sample plots (size of each plot is 0.1 ha or 31.62 × 31.62 m) nested within 0.2 ha in each site were laid out for trees enumeration. Sample size (number of *R. arboreum* tree) for a total population in each site were 166 in Phadkhal; 110 in Khirsu; 104 in Khadpatiya; 166 in Ghimtoli; 80 in Jadipani; 74 in Ranichauri; 74 in Nandasain and 96 in Nauti. Out of the standing trees in sample plots, flower bearing trees were 96 in Phadkhal; 90 in Khirsu; 102 in Khadpatiya; 126 in Ghimtoli; 64 in Jadipani; 58 in Ranichauri; 68 in Nandasain and 82 in Nauti, and without flower or smaller trees were 70 in Phadkhal; 20 in Khirsu; 02 in Khadpatiya; 40 in Ghimtoli; 16 in Jadipani; 16 in Ranichauri; 06 in Nandasain and 14 in Nauti. The individuals of all tree species in each plot were recorded along with their CBH (circumference at breast height, 1.3 m above from the ground). Individuals were categorized as mature trees (≥ 31 cm CBH), saplings (11–30 cm CBH) and seedlings (≤ 10 cm CBH)^9^. Further all the tree individuals have been grouped into 8 CBH classes: (A) 5–15 cm, (B) 16–25 cm, (C) 26–35 cm, (D) 36–45 cm, (E) 46–55 cm, (F) 56–65 cm, (G) 66–75 cm, (H) 76–85 cm. Recorded data were used for the analysis of density^10^.

**Table 1:** Physical characteristics of study sites in four districts of Garhwal region.

Sites (district)	Altitudes (m asl)	GPS location	Flower extraction surveyed villages	Distance from market (km)	Access to resources
Phadkhal (Pauri Garhwal)	1940	30º08′47″ N 78º50′46″ E	Bhattigaun	Pauri (10–12), Srinagar (25–26), Chobttakhal (2–3)	Open/easy
Khirsu (Pauri Garhwal)	1825	30º10′03″ N 78º51′47″ E	Margaun	Pauri (10–12), Srinagar (22–24), Chobttakhal (3–4)	Open/moderate
Khadpatiya (Rudrapryag)	1910	30º20′36″ N 79º03′53″ E	Kyudi	Rudrapryag (32–34), Chopta (5–6)	Open/easy
Ghimtoli (Rudrapryag)	2100	30º21′09″ N 79º05′37″ E	Ghimtoli	Rudrapryag (30–32), Chopta (10–12)	Open/easy
Jadipani (Tehri)	2270	30°24′06″ N 78°21′42″ E	Chopdiyalgaon, Nager, Saur Jadipani	Chamba (12–18), Saurjadipani (2–3)	Open/easy
Ranichauri (Tehri)	1940	30°18′13″ N 78°25′05″ E	Dargi, Sabli, Mon	Chamba (7–9), Badshahi Thol (5–7)	Open/moderate
Nandasain (Chamoli)	1820	30°11′35″ N 79°10′53″ E	Nauti, Dhhanai, Toli	Karnpryag (26–28), Nauti (3–5), Malai (1–2)	Open/moderate
Nauti (Chamoli)	1940	30°12′29″ N 79°12′39″ E	Benoli, Kaphnoli, Malai	Karnpryag (20–22), Nauti (3–5), Malai (1–2)	Open/moderate

### Flower yield estimation

Flower yield (kg/tree) was estimated during full bloom (flowering season/harvest season February–April 2017). In each sample plot, numbers of flower bearing trees varied from 29–63 trees/0.1 ha. At each site of 0.2 ha sample plot, total 40 trees, 05 flower bearing trees in each of the 08 CBH classes were marked for estimation of flower yield. The number of main branches, the number of sub- branches/offshoots per main branches (i.e. average per five randomly selected main branches per tree), and the amount of flower per sub-branches/offshoot (i.e. the average per five offshoots from the low, middle and upper canopy of each tree) were counted form marked individuals. This way flower yield/tree was calculated^9,11,12^.

The flowers from all CBH classes in each site were mixed and weighted in 5 lots of 1 kg each. The number of flowers in each lot was then counted and the mean value (400.0 ± 9.56) was considered as a standard for conversion into kilograms. Based on this conversion flower yield kg/tree was obtained. Flower yield data were pooled and mean yield (kg/tree) for each CBH class (A–H) calculated. For each site, flower yield in kg/0.2 ha was obtained by multiplying flower yield/tree by the density of flower bearing trees/0.2 ha. The total yield kg/ha for each site was calculated as *total yield* = (*yield/ha*) × *density of flower bear trees/ha*^9,11,12^.

### Extraction/harvesting and marketing trends

Flower extraction and collection were totally dependent on market availability and accessibility of site; one of the selected sites (Ranichauri) was easily accessible, while Phadkhal, Khirsu and Jadipani were moderately accessible. Khadpatiya, Ghimtoli, Nandasain and Nauti sites were far-flung from market (Table 1). The highest extraction was recorded between second week of February and first week of April. During this period, data was obtained for three consecutive days at each site.

Questionnaire based survey was carried out in selective forest fringe villages. Across the sites, total sixteen villages were selected for questionnaire survey, three villages each in Jadipani, Ranichauri, Nandasain and Nauti sites, while one village each in Phadkhal, Khirsu, Khadpatiya and Ghimtoli. In each village 15 families were randomly chosen for semi-structured questionnaire survey.

Considering the market availability for trading of the *R. arboreum* flower products, Nandasain and Nauti sites are located nearest to local market whereas Khadpatiya and Ghimtoli sites are farthest from local market. As far as the access to resources is concerned, four sites represent open and easy access to resource and four sites represent open and moderate access of resource (Table 1). During questionnaire survey, villagers were asked about the number of persons involved in resource collection (hereafter referred as collectors), age of collectors, timing of collection (early morning and late evening) etc. Ten individuals in each group (adults and children) were randomly interviewed on their harvest load to generate data on the average collection per individual, the number of days spent in flower collection, and the total income generated through this activity.

Squash/juice making factories are generally located nearby urban centers; local NGOs and small entrepreneurs are engaged in this work. These peoples purchase flower from the collectors or middleman for preparation of value product (squash). Collectors of each families (varied from *n* = 15 in Nanadasain to *n* = 31 in Jadipani) and buyers (*n* = 5 each site) were contacted to obtain information on the benefits accrued. The income values are given in Indian rupees (USD 1 = Rs. 68.00, 2017 exchange rates). Projections of potential (probable/-could generate) income (with flower processed into juice or squash) were made. The involvement of rural inhabitants as flowers collectors and the income that subsequently accrued (within a 10 km radius of fringe area) was also analyzed for sixteen villages across the sites. One adult member from each household was contacted in a village to collect information on involvement of flower collection/extraction.

### Juice/squash preparation methods and value-added products

The collected flowers are graded for their size and healthiness and the stamens are separated from petals by laborers in the juice processing unit. Petals are cleaned washed with tap water and grinded into small pieces. The petal mass is retained in the water and then boiled for one hour. The slurry (aqueous solution) obtained in this process is left at room temperature for cooling and when it get cold, filtered through linen cloth. The filtrate solution is the pure juice of the flower. For the preparation of squash from the pure juice, about 2 kg of sugar is boiled in one liter of water. Further one liter of pure juice and a small quantity of citric acid (10 g/2 kg sugar) are added to this solution. The mixture is boiled again for 30 min and then left to cool at room temperature^13^. The obtained solution known as squash is then filtered through linen cloth and stored into containers and bottles for marketing. For long term storage and good test and aroma small amount of sodium benzoate and vanilla or kawra is also mixed in the squash.

### Cost–benefit analysis of value- added products

The cost–benefit analysis of value added products prepared from the *R. arboreum* flowers was calculated in Rs./day which includes labour charges of workers involved in flower collection and materials/items required for preparation of different value added products viz: sugar, preservatives, essence, plastic containers/bottles, packaging materials etc. Labour charge was calculated on the basis of existing daily wages as per market rates. The monetary output was calculated as per the current market rates of the products (Table 2). The cost- benefit analysis of the squash product prepared from the flowers was calculated as Rs./day which includes: (i) Man days incumbent for the flowers extraction from the forest and for the preparation of squash product, (ii) Essential items such as sugar, preservatives etc. and their monetary equivalents, (iii) The total quantity of squash product and their monetary equivalents.

**Table 2:** Market cost in rupees (Rs.) of essential commodity in the preparation of *R. arboreum* juice/squash in Garhwal region.

S. no.	Name of items	Cost (Rs.)
1	Squash bottle (750 ml)	100
2	Sugar (1 kg)	45
3	Sodium benzoides (1 kg)	300
4	Citric acid (1 kg)	350
5	Plastic container (2 kg size each)	24
6	Plastic container (5 kg size each)	45
7	Plastic container (1 kg size each)	12
8	Glass bottle (750 ml)	20
9	Paraffin wax (1 kg)	350
10	Packing material	3
11	Essence/Aroma (10 ml)	12
12	Labour charge for flowers extraction (rate per day)	200

### Statistical analysis

Data failed to meet the assumption of normality (Shapiro–Wilk test) as well as homogeneity (Levene statistic); therefore, a non-parametric test (i.e. Independent–Samples Kruskal–Wallis test) was applied for one-way ANOVA. However, to find the interaction of site and cbh on flower production (yield), the same data set was subjected to two-way analysis using univariate analysis. To find if (?) flower yield depends on tree diameter or not, data of actual cbh and flower yield per tree were used to determine a correlation (Pearson Correlation Coefficient) between them. In case of correlation found significant then regression equation was developed to predict flower production based on tree diameter. All analysis were performed using IBM-SPSS 16.0 version^14^.

### Ethics approval and consent to participate

All necessary approval, free prior informed consent, permit, and certification were secured. This was done to adhere to the ethical standards of human participation in scientific research. This study was approved by Research and Consultancy Cell (Ethics Committee) of HNB Garhwal University, Srinagar Garhwal, Uttarakhand, India. All the methods were performed in accordance with the relevant guidelines and regulations.

## Results

**Figure 2:**
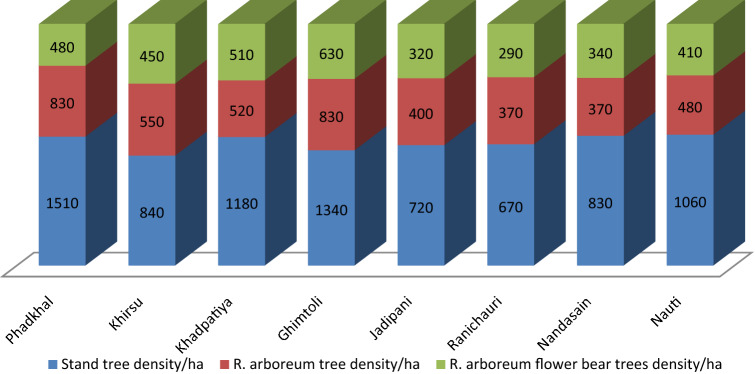
Stand tree density and *Rhododendron arboreum* density (Ind./ha) at eight sites in Garhwal region.

All eight sites have well stocked *R. arboreum* and *Q. leucotrichophora*. *Myrica esculenta, Lyonia ovalifolia, Cedrus deodara*, *Cocculus laurifolia*, *Pinus roxburghii, Quercus floribunda*, *Cornus capitata*, *Prunus cerasoides, Pyrus pashia, Abies pindrow *and* Machilus gamblei* etc. (Table 1) were the other tree species recorded from the forest sites having stand density between 670 to 1510 individuals ha^−1^. *R. arboreum* tree density varied from 370 individuals ha^−1^ (at Ranichauri and Nandasain sites) to 830 individuals ha^−1^ (at Phadkhal and Ghimtoli sites) (Fig. 2). Among the tree species, density of *R. arboreum* ranked first whereas *Q. leucotrichophora* had second rank at all sites, indicating that it is an important tree component in the forests across sites. As far as density of *R. arboreum* in particular cbh class across the sites is concerned, highest (1680 individuals ha^−1^) was recorded for 16–25 cm cbh class followed by 1110 individuals ha^−1^ in 05–15 cm cbh class and lowest 90 individuals ha^−1^ was recorded for 66–75 cm cbh class across sites.

### Flower production (yield)

**Figure 3:**
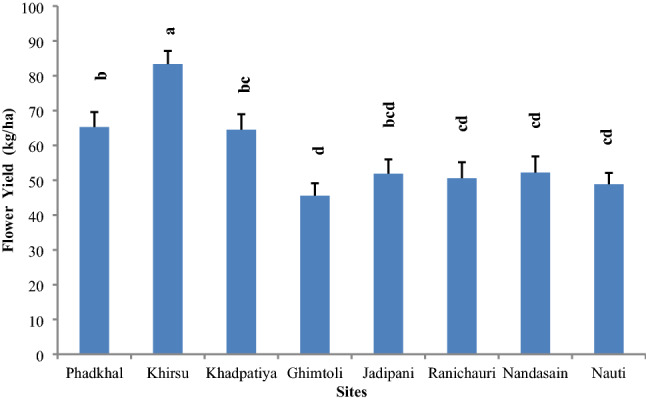
Mean flower yield (kg/ha) on *R. arboreum* trees across sites in Garhwal region. Bar with different letter indicate significant difference (Kruskal–Wallis test; H = 57.37, df = 7, p < 0.001).

Among eight study sites, highest mean flower yield (83.34 ± 3.78 kg/ha) was estimated from Khirsu site which was significantly (p < 0.001) higher than other seven sites. The lowest flower yield (45.56 ± 3.57 kg/ha) was recorded from Ghimtoli sites (Fig. 3 and Table 3). However, maximum flower bearing trees density 630 Ind./ha were recorded at Ghimtoli site followed by (510 Ind./ha) at Khadpatiya, (480 Ind./ha) Phadkhal (450 Ind./ha) at Khirsu and minimum 290 Ind./ha was recorded at Ranichauri site. It was recorded that mean flower yield was comparatively high at Khirsu, Khadpatiya and Phadkhal sites as compared to other sites, due to the availability of higher girth classes in these sites (mature class as 36–45 to 56–65 cm cbh). Mean flower yield was lower in Ghimtoli site was the result of domination of young age class (05–15 cm).

**Table 3:** Two-way ANOVA for effects of sites and cbh classes on flower yield and their interaction.

Source	Degree of freedom (df)	F value	Significance
Site	7	12.69	< 0.001
cbh	7	14.17	< 0.001
Site × cbh	49	3.36	< 0.001

**Figure 4:**
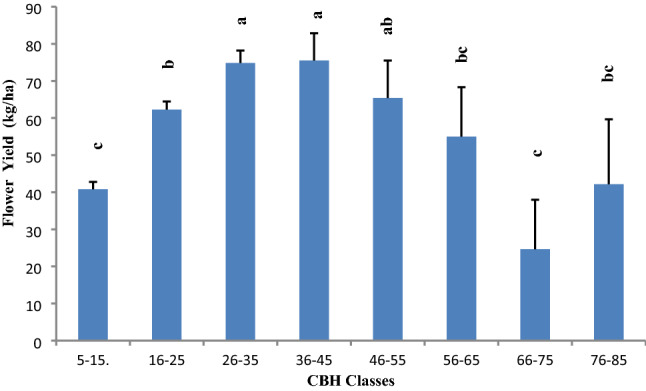
Mean flower yield (kg/ha) on *R. arboreum* trees across cbh classes in Garhwal region. Bar with different letters indicate significant difference (Kruskal–Wallis test; H = 76.75, df = 7, p < 0.001).

Based on cbh class, maximum flower bearing trees 1680 Ind./ha were recorded in 16–25 cm irrespective of site and minimum 90 Ind./ha were recorded in 66–75 cm cbh class. There was a significant variation in mean flower yield (kg/ha) with cbh classes of flower bearing trees. Mean flower yield was significantly high (p < 0.001) ranging from 65.39 ± 10.13 kg/ha to 75.49 ± 7.39 kg/ha in cbh class 26–35 to 46–55 cm, while significantly lowest flower yield 40.78 ± 1.99 kg/ha and 24.62 ± 13.37 kg/ha was recorded in cbh class of 5–15 and 66–75 cm respectively, which was closely followed by of 42.13 ± 17.54 kg/ha in cbh class of 76–85 cm (Fig. 4; Table 3). There was positively significant correlation (n = 446, p < 0.001, r = 0.53) between flower yield and actual cbh. Mean flower yield was estimated very less in class 66–75 cm as compared to other cbh class due to the number of flower bearing trees was also recorded very low in this cbh class. However, mean flower yield was estimated comparatively high in 36–46 cm and 26–35 cm cbh classes due to higher number of flower bearing trees in these classes.

### Extraction/harvesting and marketing trends

As far as flower harvesting and collection are concerned, 4 kg/adult/day or 2 kg/child/day were recorded across sites. Number of person involved for collection varied from sites and individual collected flowers for 25 days per year. Since the information was obtained in the maximum flowering harvesting period, the data analysis reflects maximum acquired harvesting. Participation of household in flower collection varies from 15 (at Nandasain site) to 31 households (at Jadipani site) and both male and females were engaged in this activity. On an average 2.9 ± 0.35 kg/trip flower collection was computed across the sites. The mean quantities of flowers harvested from forest ranged between 3.25 ± 1.23 to 29.4 ± 4.28 kg/household/year and quantities of flowers sold varied between 2.16 ± 0.98 to 20.37 ± 4.51 kg/household/year (Table 4). The numbers of squash bottle (750 ml each) prepared per year by villagers and small enterprises ranged between 10.0 ± 4.49 at Kaphnoli village in Nandasain to 92.0 ± 20.40 at Margaon village in Khirsu site. Margaon village have received maximum monetary equivalent (Rs. 9200.0 ± 2040.62 per year) with maximum net return (Rs. 3772.0 ± 836.65 per year) followed by Ghimtoli (monetary equivalent of Rs. 7800.0 ± 1025.54 and net return of Rs. 3198.0 ± 420.47). Minimum monetary equivalent (Rs. 1000.0 ± 449.32) with net return (Rs. 410.0 ± 184.22) was received by Kaphnoli village in Nandasain. The acquired and potential yield for all the sites is given in Table 5. The yield was recorded 4.61 ton/ha for Khirsu, 4.21 ton/ha for Kharpatiya, 3.91 ton/ha for Phadkhal, 3.51 ton/ha for Ghimtoli, 2.70 ton/ha for Nauti, 2.61 ton/ha for Nandasain, 2.22 ton/ha for Jadipani and 2.10 ton/ha for Ranichauri in which only small amount of existing resource (potential yield) was being extracted (8.1% for Phadkhal, 13.3% for Khirsu, 8.1% for Kharpatiya, 12.7% for Ghimtoli, 14.7% for Jadipani, 13.8% for Ranichauri, 2.8% for Nandasain and 6.1% for Nauti). This indicates the sustainable harvesting of *R. arboreum* flowers in these areas. While passing through different marketing channels, the value of the flower resources changes considerably (i.e. collector to processing unit at Rs. 200/2 kg flower; processing unit to retail market at Rs. 100/750 ml and retailer to consumer at Rs. 150/750 ml). Labour rate per day at existing market was Rs. 400. Therefore one person (male/female) harvested on an average 2 kg of fresh flowers in a half day (from 6 pm to 12 noon) so processing unit was paid for Rs. 200 per labour for 2 kg flowers. The yield potential across the sites varies from 2.10 to 4.61 ton/ha and acquired extraction among the collectors was between 0.07–0.61 ton/year which gives the income potential range from Rs. 92,000–188,000/year. Therefore, *R. arboreum* flower contributes monetary benefit of Rs. 3000–25,000/year/household from harvesting (Table 5).

**Table 4:** Mean value for flower extracted, sold, preparation of squash and average income (monetary equivalent) from *Rhododendron arboreum* in Garhwal region.

Villages/sites	Quantity extracted (kg/household/year)	Quantity sold (kg/household/year)	Number of Squash bottle (750 ml each) obtained*	Monetary equivalent (Rs.)**	Net return (Rs.)***
Bhattigaon/Phadkhal (n = 16)	19.8 ± 3.74	12.75 ± 2.86	58.0 ± 13.02	5800.0 ± 1302.88	2378.0 ± 534.18
Margaun/Khirsu (n = 21)	29.0 ± 4.28	20.37 ± 4.51	92.0 ± 20.40	9200.0 ± 2040.62	3772.0 ± 836.65
Kyudi/Khadpatiya (n = 16)	21.25 ± 1.25	16.0 ± 4.01	72.0 ± 18.55	7200.0 ± 1855.52	2952.0 ± 760.76
Ghimtoli/Ghimtoli (n = 18)	24.75 ± 2.71	19.08 ± 2.67	78.0 ± 10.25	7800.0 ± 1025.54	3198.0 ± 420.47
Chopdiyalgaon/Jadipani (n = 7)	9.78 ± 2.86	8.07 ± 2.67	36.0 ± 2.81	3600.0 ± 298.60	1476.0 ± 122.42
Nager/Jadipani (n = 12)	8.95 ± 3.07	5.9 ± 2.90	26.0 ± 14.42	2600.0 ± 1314.30	1066 ± 538.86
Saur jadipani/Jadipani (n = 12)	12.93 ± 6.81	6.68 ± 2.13	30.0 ± 9.36	3000.0 ± 969.83	1230.0 ± 397.63
Dargi/Ranichauri (n = 4)	15.37 ± 2.15	9.81 ± 1.46	72.0 ± 6.49	7200.0 ± 649.83	2935.6 ± 266.43
Sabli/Ranichauri (n = 8)	14.05 ± 3.61	7.33 ± 2.76	34.0 ± 12.61	3400.0 ± 1250.16	1394.0 ± 512.56
Mon/Ranichauri (n = 10)	9.61 ± 2.60	5.41 ± 2.01	24.0 ± 9.11	2400.0 ± 908.77	984.0 ± 372.59
Benoli/Nandasain (n = 4)	5.6 ± 0.24	3.35 ± 0.38	16.0 ± 0.58	1600.0 ± 58.46	656.0 ± 23.97
Kaphnoli/Nandasain (n = 6)	3.25 ± 1.23	2.16 ± 0.98	10.0 ± 4.49	1000.0 ± 449.32	410.0 ± 184.22
Malai/Nandasain (n = 5)	5.58 ± 1.16	3.75 ± 1.20	18.0 ± 4.93	1800.0 ± 493.99	738.0 ± 202.53
Nauti/Nauti (n = 11)	8.13 ± 1.45	3.5 ± 1.13	16.0 ± 5.08	1600.0 ± 514.12	656.0 ± 210.79
Dhhanai/Nauti (n = 9)	6.38 ± 1.25	4.05 ± 1.25	18.0 ± 2.86	1800.0 ± 286.74	738.0 ± 117.56
Toli/Nauti (n = 7)	4.00 ± 0.90	2.41 ± 0.66	12.0 ± 0.32	1200.0 ± 32.82	492.0 ± 13.46

n = number of flower extractor and seller household in village.

*1.7 Liter (1700 ml) pure juices could be obtained from one kg flowers. Calculation for production of pure juice such as mean flowers is 12.75 kg × 1700 ml juice = 21,675 ml. Convert it into number of bottle produce, capacity of one bottle is 750 ml is 21,675 ml/750 ml = 29 bottle. Two bottle squash obtained from one bottle pure juice, therefore 29 bottle pure juice produce 29 × 2 = 58 bottle squash.

**Convert it into monetary equivalent, market cost of one bottle squash is Rs. 100, therefore, 58 × 100 = Rs. 5800.

***Net return of one bottle could be Rs. 41, therefore 58 × 41 = Rs. 2378.

**Table 5:** Comparative analysis of yield potential and income acquired at different sites (yield potential refers to total flower yield in ton/ha and acquired extraction refers to total numbers of household engaged for flower extraction activities ton/year).

Site*	Yield potential (ton/ha)	Acquired extraction (ton/year)	Income potential (× Rs. 41)	Acquired net income (× Rs. 41)
Phadkhal (n = 16)	3.91	0.32	160,597.0	12,989.0
Khirsu (n = 21)	4.61	0.61	188,108.0	24,969.0
Khadpatiya (n = 16)	4.21	0.34	172,897.0	13,940.0
Ghimtoli (n = 18)	3.51	0.45	143,951.0	18,266.0
Jadipani (n = 31)	2.22	0.33	91,389.0	13,409.0
Ranichauri (n = 22)	2.10	0.31	85,034.0	11,735.0
Nandasain (n = 15)	2.61	0.07	104,796.0	2958.0
Nauti (n = 27)	2.70	0.26	112,135.0	6830.0
Total (n = 166)	25.32	2.56	1,058,907.0	105,096.0

*n = represent number of flower harvesting household.

### Cost–benefit analysis (Rs./day) and value addition

The cost–benefit analysis for juice and squash preparation from *R. arboreum* flowers is presented in Table 6. It is clearly seen that the preparation of juice and squash from the flowers is a good profitable source with a maximum per day net return of Rs. 369 per household. Across the sites, the monetary input of Rs. 200/day for labour charges including flower collection, cleaning and juice/squash extraction and total expenditure is Rs. 531. While total output is Rs. 900/day for selling of squash. It may be due to the fact that most of the large areas of Garhwal are remote where labour and transportation charges are very high. The total monetary benefit was significantly higher than the inputs for all value added items on a per day basis, therefore net return obtained per day was Rs. 369.

**Table 6:** Cost–benefit analysis (Rs./per day) of Juice/squash prepared from *R. arboreum* flower in Garhwal region.

S. no	Production measures	Monetary equivalent (Rs.)
**Input**		
1	Labour charge for flower harvesting/collection*	200
2	Labour charge for flower cleaning and juice/squash extraction	50
3	Container/bottle (750 ml)	96
4	Sugar	90
5	Preservatives (citric acid, sodium benzoate)	40
6	Essence and aroma (Vanilla kawra)	30
7	Miscellaneous (packing material)	25
Total		531
**Output**		
	Squash**	900
	Net return	369

*Two bottles (750 ml) of pure juice is obtained from one kg of fresh flowers and one person (male/female) could collect ~ 2 kg of fresh flowers per day.

**4.5 bottles (750 ml each) of pure juice is obtained from 2 kg of flowers and produce 9 bottles (750 ml each) of squash. Each bottle (750 ml) of squash could be sold for Rs. 100 in the market.

## Discussion

Khirsu, Phadkhal and Khadpatiya are the potential sites for flower production, it is evident from the presence of good number of flower bearing trees in all cbh classes, while Ghimtoli site have maximum trees in the sapling and pole stages and very few trees were observed under 26–35 to 46–55 cm cbh classes. Flower yield is much dependent on the age of trees as mature trees get more flowers than over mature and under mature trees. There was a significant variation in mean flower yield (kg/ha) among diameter class of flower bearing trees. Significantly high flower yield (p < 0.001) ranging from 65.39 ± 10.13 kg/ha to 75.49 ± 7.39 kg/ha was recorded in 26–35 to 46–55 cm cbh class respectively (Fig. 4). Flower yield and tree density in 66–100 cm gbh class was recorded better in *Pinus roxburghii* and *Cedrus deodara* forest ecotone^15^. Our result showed that flower yield was better in two cbh classes 26–35 to 46–55 cm in mixed *Q. leucotrichophora* forest ecotone. It also indicates that *R. arboreum* tree flourish well along with their natural companions (*Q. leucotrichophora*) and is sensitive to conifer forests.

**Figure 5:**
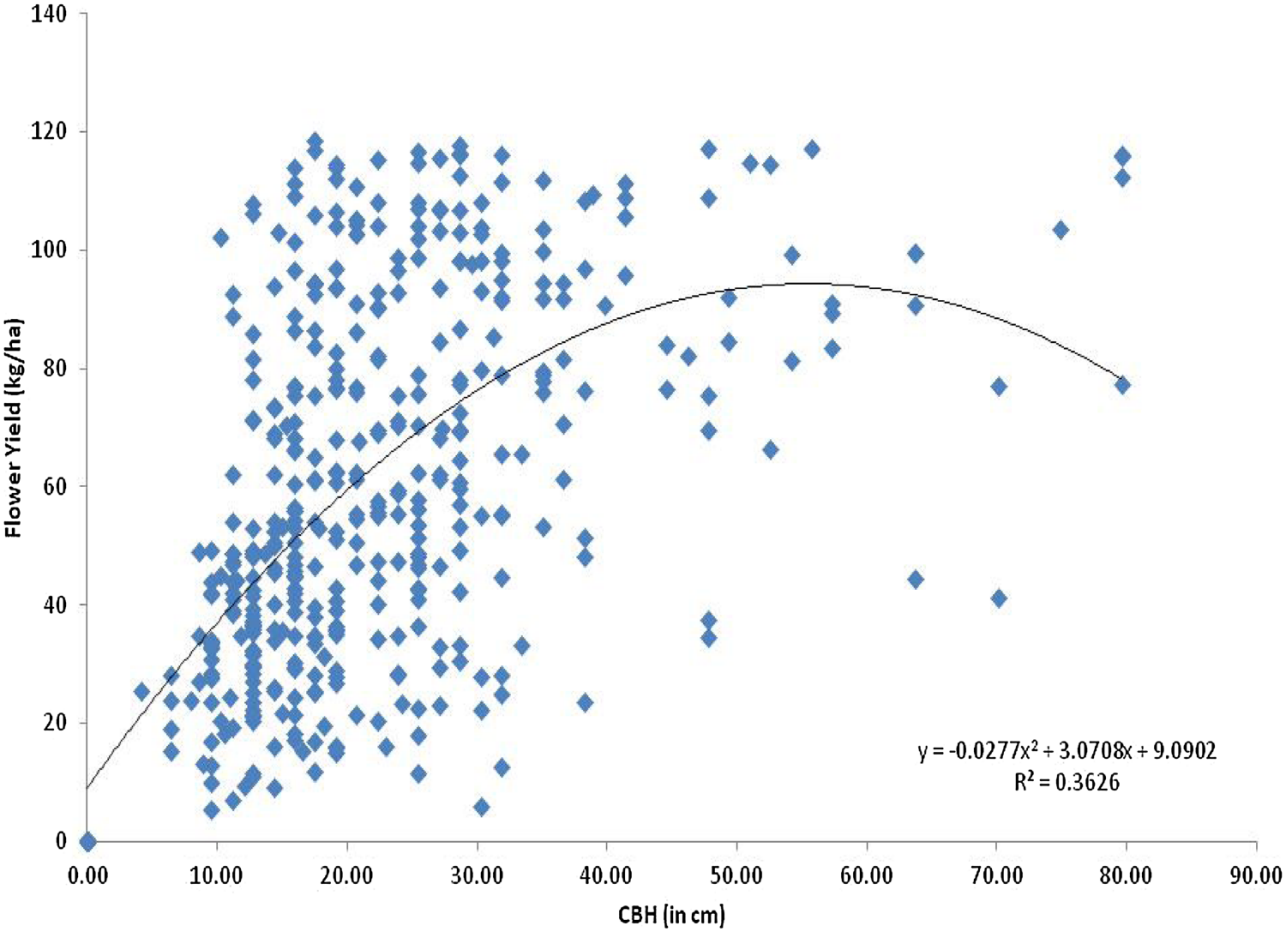
Trends of flower yield (kg/ha) with variation in tree diameter of *R. arboreum.*

Comparing the flower yield on site wise and cbh basis, it is worth to mention here that the density of flower bearing trees/ha was high at Ghimtoli site but flower yield was comparatively low as compared to Khirsu, Khadpatiya and Phadkhal sites, may be due to the fact that in Ghimtoli site most of the flower bearing trees belong to young age class (0–5 and 16–25 cm cbh classes). Moreover, flower yield was comparatively high in middle age class trees (26–35 cm and 36–46) as compared to old age class trees (66–75 cm) and young age class (Fig. 4). There was a positively significant (n = 446, p < 0.001, r = 0.53) correlation between flower yield and cbh. Based on positive and significant correlation between cbh and flower yield, a significant quadratic regression equation was developed (Y = 9.0902 + 3.0708 x – 0.0277 x^2^, R^2^ = 0.3626), which predicts 36% flower yield based on cbh (Fig. 5).

The results of present study revolved around flower extraction of *R. arboreum* from natural areas and their economic potential. Total estimated flower yield across the sites was 25.32 ton/ha and the acquired harvesting is only 10% (2.56 ton/year), thus only 10–15% resource is being used in the study area and 85–90% resource is not used. Thus a huge opportunity exists for local people to utilize this resource for strengthening their income. However, not more than 50% harvest should be allowed for resource sustainability. This shows that sustainable extraction is taking place. This situation is good in terms of sustainable use of resources as well as from regeneration point of view. Considering the conservation measures of the species, flowers extraction should be limited to 60% as sustainable manner on the single tree basis irrespective of cbh class and rest 40% of the flowers should be retained for natural regeneration^13^, thus this approach is quite good to maintaining the survival of the species in their natural habitat. It has been shown that flower yield is age dependent therefore; heavy harvesting in young as well as very old forests should not be allowed. During present study, it has been observed that people are using *R. arboreum* juice as a refreshing drink that needs to be converted into medicinal drink as the flowers have various medicinal properties as well. *R. arboreum* is a natural regenerating species and 95% of its regeneration takes place on forest floor. The forest floor is always vulnerable during harvesting period and a lot of regeneration might be destroyed at the time of harvesting of flowers. There is a need of developing harvesting protocols so that we can minimize damage during harvesting as well as sustainability of resource. The study recommend scientific harvesting techniques for sustainable productivity, capacity building of villagers on value addition, promotion of collective marketing system and promotion of *R. arboreum* as a medicinal value product.

The total estimated yield income potential is Rs. 11 lakh/year. while acquired net income is only 10% (1.1 lakh/year) from squash selling (Table 5). This study indicates that *R. arboreum* flower can contribute to cash money of stakeholders. On an average 30% of household are involved in the harvesting and trade of *R. arboreum* flower at village level, wherever resource is available. Average harvesting and collection of 25–350 kg/household/year contributes Rs. 6000–37,000 (USD 89–545)/household/year. Considering the annual per capita income in five hill districts in Garhwal region (Rudraprayag, Tehri, Uttarakashi, Pauri and Chamoli) ranges from Rs. 8352–118,448 (as against the whole state Rs. 161,102 or USD 2370) for 2016–2017, this income from *Rhododendron* is insufficient for whole year sustainability for one family^16^. In this connection, the income-producing potential of *R. arboreum* correlates well with the potential of NTFPs in India^3,9,17^.

The cost benefit analysis (Rs./day) from *R. arboreum* flower for preparation of juice and squash has good economic potential. During last few years, changes have been found in entire hill region of Uttarakhand state as *R. arboreum* flower squash has gained a good increase due to value addition. There are more than 87 villages and many NGOs and Govt. food processing centers in five hill districts of Garhwal region in Uttarakhand^18^ which are utilizing the potential of *R. arboreum* for economic benefits by preparing value addition products such as juice, squash, sauce and pickle etc. There are many rural inhabitants in this region who have chosen this business as small entrepreneur to make the livelihood during the maximum flowering season (February–April) besides engaged in various other activities of income generation. Owing to market demand and interest of general public towards herbal and nutritional food products from wild, few NGOs and stakeholders have adopted this business as a small entrepreneurship by making value added products. Local squash processing units are either supplying the products directly to shops (retail market) in nearby market and/or have created marketing network through various NGO and different trade fair organized in the state and country from time to time. Now the products are being advertised through various exhibition and fairs organized at local, district, state and national level and also being sold under the different brand name. Proper processing and selling through organized channel have enhanced market value of their products and these platforms enable them to access quicker benefit.

There is good scope of *R. arboreum* flowers for preparation of quality value added products and developing small entrepreneur at village level to serve the purpose of employment and income generation for sustaining the local people. Uttarakhand, particularly Garhwal region is an great significance spiritual (Char dham) and tourist place, millions of pilgrims visit these places every year which make existing the market demand of the product very high. Numbers of entrepreneurs have linked their business to the eco-tourism and gaining high economic benefit through marketing of this produces during peak tourist seasons. Therefore, if the effective promotion is carried out, their market demand will increase rapidly. Presently the economic benefit driven from Juice leads to more interest towards the species; people along with government should go for participatory conservation of the species. Obviously, the people will be aware of the importance of the species while getting the economic benefit and certainly *R. arboreum* will be conserved by the people themselves. Since the *R. arboreum* are keystone species thus making conservation of the species more is important. Due to poor regeneration and anthropogenic pressure on the species, flower extraction should be limited to 60% on the single tree basis irrespective of cbh class and rest 40% of the flowers should be left out on tree to mature into seed for conservation measures. Flowers should be harvested by climbing the trees without cutting down the branches. Finally within the context of ecological and economic sustainability, the extraction, yield and economic potential of *R. arboreum* in these areas is significant. There is no danger of depletion considering present flowers extraction methods.

## Data Availability

All data generated or analyzed during this study are included in this published article.
